# Inference of dynamic biological networks based on responses to drug perturbations

**DOI:** 10.1186/s13637-014-0014-1

**Published:** 2014-09-25

**Authors:** Noah Berlow, Lara Davis, Charles Keller, Ranadip Pal

**Affiliations:** 1 Department of Electrical and Computer Engineering, Texas Tech University, Lubbock, 79409 TX USA; 2 Department of Pediatrics, Oregon Health & Science University, Portland, 97239 OR USA

**Keywords:** Drug perturbation experiments, Network inference, Pathway design

## Abstract

Drugs that target specific proteins are a major paradigm in cancer research. In this article, we extend a modeling framework for drug sensitivity prediction and combination therapy design based on drug perturbation experiments. The recently proposed target inhibition map approach can infer stationary pathway models from drug perturbation experiments, but the method is limited to a steady-state snapshot of the underlying dynamical model. We consider the inverse problem of possible dynamic models that can generate the static target inhibition map model. From a deterministic viewpoint, we analyze the inference of Boolean networks that can generate the observed binarized sensitivities under different target inhibition scenarios. From a stochastic perspective, we investigate the generation of Markov chain models that satisfy the observed target inhibition sensitivities.

**Electronic supplementary material:**

The online version of this article (doi:10.1186/s13637-014-0014-1) contains supplementary material, which is available to authorized users.

## 1Introduction

Personalized medicine based on individual genetic circuit is a primary goal of Systems Medicine research. The application of a population-averaged pathway for an individual cancer patient limits the success of targeted therapies since there can be huge variations in the regulatory pathways of distinct cancer patients [1]–[5]. Generating a detailed model of the specific regulatory pathway of the patient is extremely difficult due to the enormous experimental data requirements on model parameter estimation. Often, only a specific aspect of the regulatory system is considered based on the final objective of modeling. For instance, the goal of individual tumor sensitivity to targeted drugs is frequently based on genetic mutations [6], gene expression measurements [7], or a combination of genetic and epigenetic information [8],[9]. The approach of using genetic mutations for predicting the sensitivity is restricted by the presence of non-functional mutations and other latent variables. Statistical tests have been used to show that genetic mutations can be predictive of the drug sensitivity in non-small cell lung cancers [6], but the classification rates for the aberrant samples are still low. In [7], gene expression profiles are used to predict the binarized efficacy of a drug over a cell line with the accuracy of the designed classifiers ranging from 64*%* to 92*%*. In [10], a co-expression extrapolation (COXEN) approach was used to predict the drug sensitivity for samples outside the training set with an accuracy of around 80%. [8] uses Elastic Net modeling over multiple genetic characterizations to achieve Pearson correlation coefficients in the range of 0.1 to 0.8 between experimental and predicted drug sensitivities. [11] has used Random Forests over the NCI 60 cancer cell lines for drugs sensitivity prediction. Tumor sensitivity prediction has also been considered as (a) a drug-induced topology alteration [12] using phospho-proteomic signals and prior biological knowledge of generic pathway and (b) a molecular tumor profile-based prediction [6],[13].

We have considered a functional approach based on tumor cell viability to multiple kinase inhibitor drugs [14],[15]. The experimental data is generated using a drug screen consisting of *D* multi-target kinase inhibitor compounds and subjecting tumor cells to this array. Sensitivity for the individual drugs is measured after 72 h. The model developed from this approach is able to predict the steady state behavior of target inhibitor combinations but does not provide us with the dynamics of the model or the directionality (upstream or downstream) of the inferred target blocks.

In this article, we analyze the generation of possible dynamic models satisfying the steady state model representation. We first show that the Target Inhibition Map (TIM) [14],[15] approach can generate blocks of targets that are connected in series to form a pathway but the directionality of the blocks are unknown. Subsequently, we establish that a directional pathway can be converted to a deterministic Boolean network (BN) [16] model. The discrete representation of the TIM as a directional pathway allows us to select a minimal number of sequential inhibition experiments for inferring the actual dynamic model. To incorporate the continuous sensitivity behavior following drug inhibition, we consider the inverse problem of generation of Markov chains that satisfies for every target inhibition condition: the steady-state probability of non-tumorous state is equal to the normalized sensitivity. The set of dynamic models producing the static TIM can be utilized for robustness analysis of the combination therapy design and design of time-dependent combination therapies. The approach presented in this paper extends the static design to incorporate possible dynamics.

The paper is organized as follows. Section 2 provides a brief description of the TIM approach; Section 3 describes inference of deterministic BNs from TIM. The generation of stochastic Markov chains based on the TIM is presented in Section 4, and the conclusions are included in Section 5.

## 2Target inhibition map model

**Figure 1:**
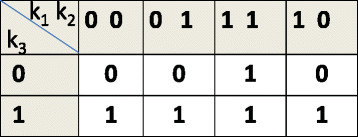
**TIM for mutations in** ***K***_***1***_ **and** ***K***_***2***_ **.**

In a recently proposed approach (details available in [14],[15]), we considered experimental data on tumor sensitivity for various target inhibition combinations (corresponding to different multi-target inhibitory drugs) and generated a TIM model. The TIM predicts the steady-state tumor phenotypes for binary combinations of inhibition of functionally relevant targets (i.e. for *n* targets, there will be 2^*n*^ possible inhibition combinations). An example TIM for three targets *K*_1_,*K*_2_,*K*_3_ is shown in Figure 1. The map in Figure 1 shows that inhibition of *K*_3_ alone can inhibit the tumor or inhibition of both *K*_1_ and *K*_2_ can inhibit the tumor. The current setting of the TIM approach will consider only those targets that are functionally relevant in cell death in a new cancer sample. These targets are often up-regulated in cancer either due to their own mutations or activations by some other enzymes (from now onwards, we will call such activations by enzyme(s) not considered in the final TIM as *latent activations*). The TIM approach has also been extended to model continuous scaled sensitivity predictions, i.e., the steady state predictions for various binary target inhibition combinations will be in the range [ 0,1].

We should note that the TIM only provides a steady state snapshot of the regulatory behavior occurring in a cancer pathway following application of various target inhibitors. The TIM can be used to arrive at possible infinite horizon simultaneous combination therapies with fixed intervention at all-time steps. The next step in the framework is exploring the possible dynamical models producing the steady state TIM. The advantages of exploring the dynamics of the TIM include (a) *model-based experimentation,* having a constrained set of dynamical models that can produce the TIM will allow us to algorithmically generate the optimal set of target expression measurements required to decipher the actual unique dynamical model, and (b) *sequential drug delivery*, the dynamical models can be used to analyze the behavior of sequential combination drug application.

## 3Discrete deterministic dynamic model inference

The primary contribution of the paper lies in the generation of stochastic Boolean network models satisfying the given normalized sensitivities for different inhibition combinations. We consider the generation of the Markov models based on altering the deterministic dynamical models. In this section, a review of the work generating discrete deterministic dynamical models reported in [14] is presented to enhance the readability of the subsequent section on inference of stochastic dynamical models.

**Figure 2:**
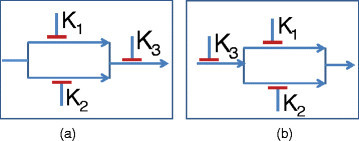
**Possible directional pathways based on the TIM in Figure** 1 **(a, b).**

To arrive at potential discrete deterministic dynamical models, we consider the likely directional pathways that can generate the inferred TIM and map the directional pathways to deterministic BN models. The TIM can be used to locate the feasible mutation patterns and constrain the search space of the dynamic models generating the TIM. For instance, mutation or external activation of *K*_2_ or *K*_1_ alone cannot result in the TIM of Figure 1; otherwise, the inhibition of *K*_2_ or *K*_1_ should have been able to block the tumor. Thus, feasible mutations or latent activation patterns are reduced to the following five sets of combinations, {*K*_1_,*K*_2_},{*K*_1_,*K*_3_},{*K*_2_,*K*_3_},{*K*_3_},{*K*_1_,*K*_2_,*K*_3_}, out of possible eight combinations. For each mutation or latent activation pattern, we can arrive at possible directional pathways producing the required steady state TIM output. For instance, Figure 2 shows two directional pathway possibilities for mutation or activation patterns {*K*_1_,*K*_2_} and {*K*_3_}, respectively. The pathways in Figure 2 show possible tumor survival circuits. In this model, if a left-to-right tumor survival pathway exists, the cancer survives. If the path is stopped, the tumor cells stop growing or involute.

### 3.1 3.1 Optimal set of experiments to infer the directional pathway structure

**Figure 3:**
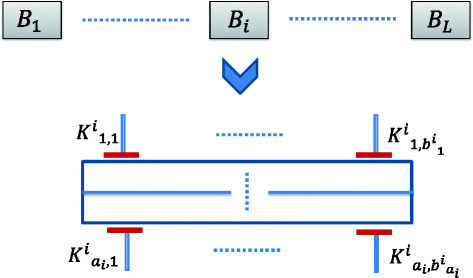
**A general abstract pathway resulting from a TIM.**

In this subsection, we analyze the minimum number of expression measurement experiments required to decipher the pathway directionality once the steady state structure (TIM) has been inferred. Knowledge of target expressions can be used to narrow down the possible directional pathways. For instance, expressed *K*_1_ following inhibition of *K*_3_ for our earlier example will denote the feasibility of directional pathway of Figure 2a and removing the possibility of the directional pathway shown in Figure 2b. Note that latent activations and functionally irrelevant mutations may restrict the usefulness of mutation status in restricting the pathway search space. In the following paragraphs, we will consider a general pathway obtained from a TIM having the structure shown in Figure 3 but with unknown directionalities of the blocks and target positions. We will consider that the pathway has *L* blocks in series (*B*_1_,*B*_2_,⋯,*B*_*L*_) and each block *B*_*i*_ has *a*_*i*_ parallel path segments with each segment *j* containing $b_{j}^{i}$ targets $\left(K_{1,1}^{i},K_{1,2}^{i}\cdots \;,K_{1,b_{j}^{i}}^{i}\right)$. The total number of targets in the general map is $N_{K}=\overset{L}{\underset{i=1}{\sum }}\overset{a_{i}}{\underset{j=1}{\sum }}b_{j}^{i}$.

Assuming that the *N*_*K*_ targets are distinct, the maximum number of distinct discrete dynamic models satisfying the structure is $L!\overset{L}{\underset{i=1}{\prod }}\overset{a_{i}}{\underset{j=1}{\prod }}(b_{j}^{i})!$. If Figure 3 represents a possible directional orientation, the only targets that will have initial activations for the target inhibition combination $K_{1,1}^{1},K_{2,1}^{1}\cdots K_{a_{1},1}^{1}$ to be effective is $K_{1,1}^{1},K_{2,1}^{1}\cdots K_{a_{1},1}^{1}$. For our analysis, we are assuming that we can inhibit specific targets of our choice and we can measure the steady state target expression following application of the target inhibitions. We can locate the directionality of the blocks *B*_1_ to *B*_*L*_ with respect to each other (downstream or upstream) with the worst-case scenario of *L*−1 steady state measurements. The expected number of experiments required to detect the directionality of *L* serial blocks is $\frac{2L-1}{3}$ for *L*≥2. To infer the directionality of targets in each parallel line of the block, one target from each line up to a maximum of *a*_*i*_−1 lines will be inhibited for each block *B*_*i*_. If we consider a single block *B*_*i*_, each experiment can detect the location of *a*_*i*_−1 targets; thus, the total number of experiments required to decipher the possible directionalities (upstream or downstream) of the targets in the block *B*_*i*_ is $\leq \max\left(\underset{j\in S_{i}}{\max}\overset{i}{\underset{j}{b}}-2,\lceil \frac{\underset{j\in S_{i}}{\sum }\overset{i}{\underset{j}{b}}-a_{i}}{a_{i}-1}\rceil -1\right)$ where *S*_*i*_={1,⋯,*a*_*i*_}. Thus for the overall map, the worst case number of experiments $N_{E}^{w}$ required to decipher the directionalities of all the targets is upper-bounded by [17]

1 $$\;\begin{array}{c} N_{E}^{w}\leq \underset{i\in S}{\max}\left\{\max\left(\underset{j\in S_{i}}{\max}\overset{i}{\underset{j}{b}}-2,\lceil \frac{\underset{j\in S_{i}}{\sum }\overset{i}{\underset{j}{b}}-a_{i}}{a_{i}-1}\rceil --1\right)\right\}+L-1 \end{array}$$

where *S*={1,⋯,*L*}. The expected number of experiments $N_{E}^{a}$ required to decipher the directionalities of all the targets is upper-bounded by

2 $$\;\begin{array}{c} N_{E}^{a}\leq \underset{i\in S}{\max}\left\{\max\left(\underset{j\in S_{i}}{\max}\frac{2\overset{i}{\underset{j}{b}}-4}{3},\lceil \frac{\underset{j\in S_{i}}{\sum }2\overset{i}{\underset{j}{b}}-a_{i}}{3(a_{i}-1)}\rceil --1\right)\right\}+\frac{2L-1}{3} \end{array}$$

#### 3.1.1 3.1.1 Simulation results on optimal experimental steps

**Figure 4:**
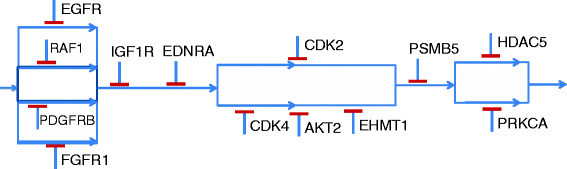
**Pathway derived from perturbation experiments.**

**Figure 5:**
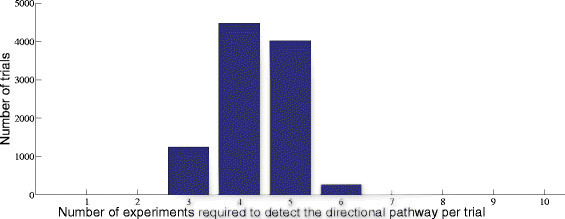
**Distribution of number of target expression measurements.**

For our simulation results, we consider a pathway derived from targeted drug perturbation experiments carried out at Keller Laboratory at Oregon Health and Science University on canine osteosarcoma cell cultures. Sixty targeted cancer drugs were tested on cell cultures, and a TIM was generated based on the viability data using the approach provided in [14],[15]. For our simulation results, we will consider one of the plausible directional pathways derived from the TIM to be the actual pathway and estimate the number of target expression measurements required to arrive at it if the directional information is not known. The directional pathway assumed to be the actual pathway is shown in Figure 4 consisting of 13 targets. If we compare Figure 4 with the general pathway in Figure 3, the number of serial blocks *L*=6. Similarly, *a*_1_=4,*a*_2_=1,*a*_3_=1,*a*_4_=2,*a*_5_=1,*a*_6_=2, and $b_{j}^{i}=1$ for all *i* and *j* except $b_{2}^{4}=3$. Since there is only one serial block with $b_{j}^{i}>2,$ we can reduce Equation 1 to $N_{E}^{w}\leq \underset{i\in S,j\in S_{i}}{\max}b_{j}^{i}-2+L-1=6$ and Equation 2 to $N_{E}^{a}\leq \underset{i\in S,j\in S_{i}}{\max}\frac{2b_{j}^{i}-4}{3}+\frac{2L-1}{3}=4.33$. To compare these numbers with simulation results, we conducted 10,000 simulation runs to detect the pathway shown in Figure 4 starting from random inhibition of serial blocks. The distribution of the number of steady state experiments required to detect the directional pathway is shown in Figure 5. We note that the maximum number of experiments required was 6 as given by $N_{E}^{w}$ in Equation 1, and the expectation of the distribution is 4.33 which is the same as the bound on $N_{E}^{a}$ given by Equation 2.

### 3.2 3.2 Deterministic dynamical model from directional pathway

To generate a BN model of a directional pathway, we will first consider the starting mutations or latent activations. The number of states in the BN will be 2^*n*+1^ for *n* targets. Each state will have *n*+1 bits with first *n* bits referring to the discrete state of the *n* targets and the least significant bit (LSB) will correspond to the binarized phenotype, i.e., tumor (1) or normal (0).

The rules of state transition for this special class of BNs are as follows [17]: *Rule a*. A target state at time *t*+1 becomes 1 if any immediate upstream neighbor has state 1 at time *t* for OR relationships or all immediate upstream neighbors have state 1 at time *t* for AND relationships. Note that the examples have OR type of relations as they are the most commonly found relations in biological pathways (based on illustrated pathways in KEGG). *Rule b*. For the BN without any drug, the targets that are mutated or have latent activations will transition to state 1 within one-time step. *Rule c.* For a target with no inherent mutation or latent activation, the state will become 0 at time *t*+1 if the immediate upstream activators of the target has state 0 at time *t*.

The BN construction from directional pathways mentioned above is described for targets acting as oncogenes (activation causing cancers), but it can also be extended to tumor suppressors (inhibition causing cancers) by considering the inverse state of the tumor suppressor in the above framework.

**Figure 6:**
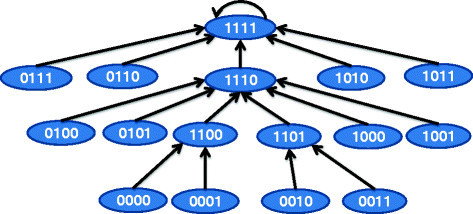
**State transitions of the BN for the directional pathway in Figure** 2 **a.**

We illustrate the BN construction algorithm using the example of the pathway shown in Figure 2a. The downstream target *K*_3_ can be activated by either of the upstream activated targets *K*_1_ or *K*_2_. The corresponding BN transition diagram for this pathway is shown in Figure 6. For instance, if we consider the state 1001 at time *t*, it denotes *K*_2_, *K*_3_ being inactive and *K*_1_ being active and the phenotype being tumorous. Based on the directional pathway in Figure 2a, tumor proliferation is caused by activated *K*_3_ and thus the phenotype will change to non-tumorous (i.e., 0) at *t*+1. The activated *K*_1_ will activate *K*_3_ at time *t*+1 and *K*_2_ will also be activated in the absence of continued inhibition as we assumed that mutation or latent activations activate both *K*_1_ and *K*_2_. Thus, the next state at time *t*+1 will be 1110. Note that we are considering that the effect of one application of the drug remains for one-time step and thus the targets *K*_1_ and *K*_2_ revert back to 1 if the drug is not continued in the next time step. If the drug effect continues for multi-time steps, then 1001 will transition to 1010. Note that some transitions may appear like the tumor state is oscillating in the transient phase such as the path 0010→1101→1110→1111. The reason is that the network can only be in the starting state 0010 where *K*_1_ and *K*_2_ is inactivated through application of some external intervention and not through normal transitions as the network has *K*_1_ and *K*_2_ mutated. Scenarios following application of drugs can produce alternating tumor proliferation and inactivation states in the transient phase.

### 3.3 3.3 Altered BN following target inhibition

**Figure 7:**
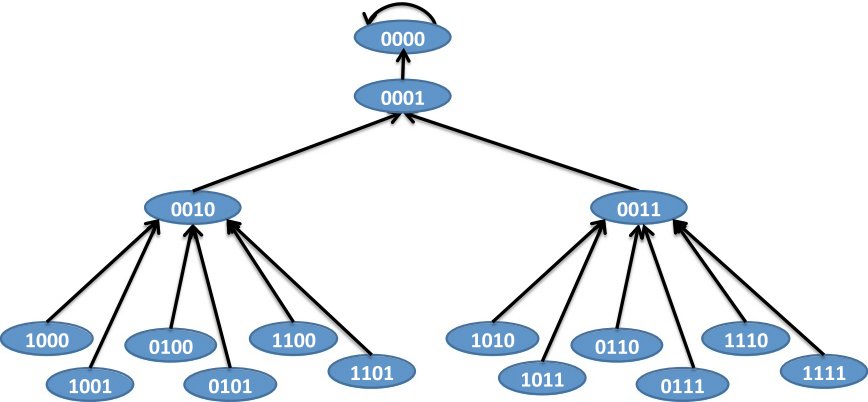
**BN state transition following inhibition of targets** ***K***_***1***_ **and** ***K***_***2***_ **.**

The BN in Figure 6 can also be represented by a 16×16 transition matrix *P* representing the state transitions. To generate the dynamic model after inhibition of *s* specific targets *I*={*K*_1_,*K*_2_,⋯,*K*_*s*_} (by application of targeted drugs), the transition *i*→*j* in the untreated system will be converted to *i*→*z* in the treated system where *z* is *j* with targets *I* set to 0. Each target inhibition combination can be considered as multiplying the initial transition matrix *P* by an intervention matrix *T*_*c*_. Each row of *T*_*c*_ contains only one non-zero element of 1 based on how the inhibition alters the state. If we consider *n* targets, *n* *T*_*c*_’s in combination can produce a total of 2^*n*^ possible transformation matrices $T_{1},T_{2},\cdots \;,T_{2^{n}}$. The TIM denotes the state of the LSB of the attractor for the 2^*n*^ transition matrices ${\text{PT}}_{1},{\text{PT}}_{2},\cdots \;,{\text{PT}}_{2^{n}}$ starting from initial state 11⋯1 (i.e., all targets considered in the TIM and tumor are activated). For instance, if we consider that our drug inhibits the targets *K*_1_ and *K*_2_ (i.e., set *S*_1_={*K*_1_,*K*_2_}), the discrete dynamic model following application of the drug is shown in Figure 7. The intervention matrix corresponding to the inhibition of *K*_3_ is shown in Table 1. The transition *i*→*j* is 1 only when inhibition of the first and second bits of *i* results in *j*.

**Table 1:** **Inhibition matrix** ***T***_***c***_ **for inhibition of** ***K***_***1***_ **and** ***K***_***2***_

	0 0 0 0	0 0 0 1	0 0 1 0	0 0 1 1	0 1 0 0	0 1 0 1	0 1 1 0	0 1 1 1	1 0 0 0	1 0 0 1	1 0 1 0	1 0 1 1	1 1 0 0	1 1 0 1	1 1 1 0	1 1 1 1
0 0 0 0	1	0	0	0	0	0	0	0	0	0	0	0	0	0	0	0
0 0 0 1	0	1	0	0	0	0	0	0	0	0	0	0	0	0	0	0
0 0 1 0	0	0	1	0	0	0	0	0	0	0	0	0	0	0	0	0
0 0 1 1	0	0	0	1	0	0	0	0	0	0	0	0	0	0	0	0
0 1 0 0	1	0	0	0	0	0	0	0	0	0	0	0	0	0	0	0
0 1 0 1	0	1	0	0	0	0	0	0	0	0	0	0	0	0	0	0
0 1 1 0	0	0	1	0	0	0	0	0	0	0	0	0	0	0	0	0
0 1 1 1	0	0	0	1	0	0	0	0	0	0	0	0	0	0	0	0
1 0 0 0	1	0	0	0	0	0	0	0	0	0	0	0	0	0	0	0
1 0 0 1	0	1	0	0	0	0	0	0	0	0	0	0	0	0	0	0
1 0 1 0	0	0	1	0	0	0	0	0	0	0	0	0	0	0	0	0
1 0 1 1	0	0	0	1	0	0	0	0	0	0	0	0	0	0	0	0
1 1 0 0	1	0	0	0	0	0	0	0	0	0	0	0	0	0	0	0
1 1 0 1	0	1	0	0	0	0	0	0	0	0	0	0	0	0	0	0
1 1 1 0	0	0	1	0	0	0	0	0	0	0	0	0	0	0	0	0
1 1 1 1	0	0	0	1	0	0	0	0	0	0	0	0	0	0	0	0

**Figure 8:**
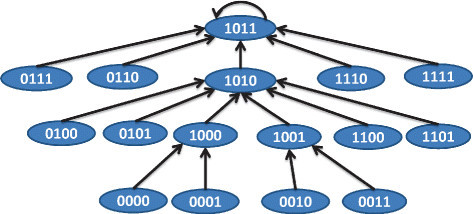
**BN state transitions following inhibition of target** ***K***_***2***_ **.**

We should note that the equilibrium state of the network 0000 has 0 for the tumor state. This is because the tumor is activated by *K*_3_ and inhibition of *K*_1_ and *K*_2_ blocks activation of *K*_3_ and thus should eradicate the tumor. On the other hand, since both *K*_1_ and *K*_2_ can cause tumor through activation of intermediate *K*_3_, inhibition of only one of *K*_1_ and *K*_2_ will not block the tumor. The BN following inhibition of *K*_2_ is shown in Figure 8 where the attractor 1011 denotes a tumorous phenotype.

## 4Discrete stochastic dynamic model inference

**Figure 9:**
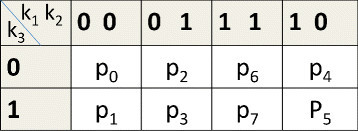
**A probabilistic TIM.**

The analysis so far has considered deterministic discrete binary states for the targets and tumor phenotype. A stochastic modeling approach will be preferred when we want to take into consideration that tumor phenotype (measured in terms of tumor size reduction, *I* *C*_50_ or cell cycle arrest) is a continuous variable. We have extended our TIM approach to probabilistic target inhibition map (PTIM) where the PTIM provides continuous sensitivity prediction values between 0 to 1 for all possible kinase inhibition combinations [14],[15]. From a stochastic dynamical model perspective, we can consider the sensitivity prediction value provided by the PTIM as the steady state probability of the tumor phenotype being 0 (a similar approach with deterministic differential equation models for modeling the tumor sensitivity was considered in [18] and experimental data was assumed to reflect the steady state values). For instance, if we consider that a Markov chain of 16 states explain our dynamical model for the pathway shown in Figure 9, the entry PTIM (i, j) will reflect the steady state probability for the LSB = 0 for the model with target inhibitions *i*,*j*. For instance, *p*_5_ reflects the sensitivity with target inhibition *K*_1_ and *K*_3_.

In this article, the discrete stochastic dynamic behavior will be modeled by a Markov chain where the states of the Markov chain contain information on the protein expressions of the targets and the tumor status. Note that a detailed stochastic master equation model is a continuous time Markov chain and can be approximated by a discrete time Markov chain based on a suitable time step [19]. Also, Boolean networks can be incorporated as Markov chains where each row of the transition probability matrix contains a single 1 with remaining all entries being 0.

For the subsequent analysis, we will consider that we have *n* binarized targets in our model and the states of the Markov chain will be 0⋯0 to 1⋯1 where the LSB will denote the state of the tumor (1 denoting tumor proliferation and 0 denoting tumor reduction) and the remaining *n* bits denote the state of the *n* targets. The set of states of the Markov chain, denoted by the set , is of size *N*=2^*n*+1^. Let *P* denote the *N*×*N* transition probability matrix. Let *f*_*c*_(*j*) denote the value of the state *j* following Boolean intervention equal to the inverse binary value of decimal *c*, i.e., under intervention *f*_3_(*x*)=*x* AND (0011) denoting intervention of first and second targets and *f*_5_(*x*)=*x* AND (0101) denoting intervention of first and third targets. Let $I_{c}$ denote the possible set of states following application of intervention *c*, i.e., $I_{c}$ contains only the states *i* s.t. *f*_*c*_(*i*)=*i*. For the above example, $I_{3}=\{0000,0001,0010,0011\}$. Let *S*_*c*,*i*_ denote the set of states *j* for which *i*=*f*_*c*_(*j*). Here, *S*_3,0_={0000,1000,0100,1100}, the set of states which, under inhibition *f*_3_(·), transition into state *i*.

The targeted drugs usually inhibit a set of target proteins and modeling such a behavior can be approached in one of the two following ways: (A _*i*_) If the targeted drugs inhibit the set of proteins *I*, the dynamics of the system under drug delivery can be considered as a new Markov chain with transition probability matrix *P*_2_ where the *j* th row of *P*_2_ is same as the *i* th row of *P*, where *j* is *i* with targets *I* set to 0. For instance, in a four-target system where *I* is targets 1 and 2, rows 0000, 1000, 0100, and 1100 of *P*_2_ will be the same as row 0000 of *P*. This approach refers to resetting the system to the state obtained by applying the drug and let it evolve from there. Note that the above described system will still show non-zero transition probabilities to states where the target set *I* may have non-zero values. (A _*ii*_) If we have transition probability *P*(*i*,*j*) of moving from state *i* to state *j* in the uncontrolled system, for the new system with transition probability matrix *P*_3_, we will add the transition probability *P*(*i*,*j*) to *P*(*i*,*z*) where *z* is *j* with targets *I* set to 0.This encompasses the behavior that if the system transitions from *i* to *j*, *j* has been turned to *z* by the intervention.

The following theorem proves that the aggregated steady state probability distribution for both the approaches are equal.

### **Theorem4.1**.

Let *π*_2_ and *π*_3_ denote the stationary probability distributions of *P*_2_ and *P*_3_. If $\pi_{2}^{*}$ denotes the aggregration of states after intervention *C*, i.e., $\pi_{2}^{*}(i)=\underset{i_{1}\in S_{i,c}}{\sum }\pi_{2}(i_{1})$ for $i\in I_{C}$ and $\pi_{2}^{*}(i)=0$ for $i\notin I_{C}$, then $\pi_{2}^{*}$ also satisfies the stationary probability distribution equations for *P*_3_, i.e., $\pi_{2}^{*}=\pi_{2}^{*}P_{3}$. If P is ergodic, then $\pi_{2}^{*}=\pi_{3}$.

### *Proof*.

Let *f*_*c*_(·) be a Boolean intervention function. We have ∀i,j∈I

3 $$P_{2}(i,j)=P(f_{c}(i),j)$$

and $P_{3}(i,j)=\underset{k\in S_{c,j}}{\sum }P(i,k)$. The stationary distribution for *P*_2_ will satisfy

4 $$\begin{array}{lcr} \;\pi_{2}(i) & = & \underset{j\in I}{\sum }\pi_{2}(j)P_{2}(j,i)\\ = & \underset{z\in I_{C}}{\sum }P(z,i)\underset{k\in S_{c,z}}{\sum }\pi_{2}(k) \end{array}$$

Similarly, the stationary distribution for *P*_3_ will satisfy *π*_3_(*i*)=0 for $i\notin I_{C}$ and for $i\in I_{C}$:

5 $$\begin{array}{lcr} \;\pi_{3}(i) & = & \underset{j\in I}{\sum }\pi_{3}(j)P_{3}(j,i)\\ = & \underset{z\in I_{C}}{\sum }\pi_{3}(z)P_{3}(z,i)\\ = & \underset{z\in I_{C}}{\sum }\pi_{3}(z)\underset{k\in S_{i}}{\sum }P(z,k) \end{array}$$

If $\pi_{2}^{*}$ denotes the aggregration of states, i.e., $\pi_{2}^{*}(i)=\underset{i_{1}\in S_{c,i}}{\sum }\pi_{2}(i_{1})$ for $i\in I_{C}$, then we have for $i\in I_{C}$:

6 $$\begin{array}{lcr} \;\pi_{2}^{*}(i)=\underset{k\in S_{c,i}}{\sum }\pi_{2}(k) & = & \underset{k\in S_{c,i}}{\sum }\underset{z\in I_{C}}{\sum }P(z,k)\underset{j\in S_{z}}{\sum }\pi_{2}(j)\\ = & \underset{z\in I_{C}}{\sum }\underset{k\in s_{c,i}}{\sum }P(z,k)\underset{j\in S_{c,z}}{\sum }\pi_{2}(j)\\ = & \underset{z\in I_{C}}{\sum }\pi_{2}^{*}(z)\underset{k\in S_{c,i}}{\sum }P(z,k) \end{array}$$

Comparing Equations 5 and 6, we note that $\pi_{2}^{*}$ also satisfies the stationary probability distribution equation for *P*_3_, i.e., $\pi_{2}^{*}P_{3}=\pi_{2}^{*}$.

We will model the target intervention based on perspective *A*_*i*_. We next analyze whether every PTIM can be represented by a Markov chain. Theorem 4.2 shows that there always exists a Markov chain construction that can satisfy the PTIM steady state sensitivities.

### **Theorem4.2**.

For any given PTIM, ∃ at least one Markov chain satisfying the PTIM.

### *Proof*.

Consider a PTIM with *n* targets *K*_1_,*K*_2_,⋯,*K*_*n*_ and thus 2^*n*^ PTIM entries $p_{0},p_{1},\cdots p_{2^{n}-1}$, where *p*_*i*_ denotes the PTIM-predicted steady state probability of tumor reduction when the active targets in the binary representation of *i* are inhibited. Denote the treatments corresponding to each *p*_*i*_ as *g*_*i*_. A trivial Markov chain satisfying the PTIM can be generated as follows: ∀*i*∈[0,⋯,2^*n*^−1], we can generate a unique pair of *n*+1 dimensional states *D*_1_=2(2^*n*^−*i*−1) and *D*_2_=2(2^*n*^−*i*−1)+1. *D*_1_ and *D*_2_ differ only in the last bit indicating tumor proliferation status. Here, LSB of *D*_1_=0 and LSB of *D*_2_=1. The first *n* bits of the binary representation of *D*_1_ and *D*_2_ are 0 where the representation of *i* has value 1. Consider a 2^*n*+1^×2^*n*+1^ Markov chain with transition probability matrix *P*. ∀*i*∈[0,⋯,2^*n*^−1], let us assign probabilities as *P*(*D*_1_,*D*_1_)=*p*_*i*_, *P*(*D*_1_,*D*_2_)=1−*p*_*i*_, *P*(*D*_2_,*D*_1_)=*p*_*i*_, and *P*(*D*_2_,*D*_2_)=1−*p*_*i*_. This particular Markov chain will satisfy our given PTIM. Since, there are 2^*n*^ closed classes of 2 states each, the stationary probability for inhibiting *i* can be calculated from considering the steady state probabilities of the Markov chain

$$\left|\begin{array}{ll} p_{i} & 1-p_{i}\\ p_{i} & 1-p_{i} \end{array}\right|$$

which is *p*_*i*_ for the state with tumor = 0 and 1−*p*_*i*_ for the state with tumor = 1.

### 4.1 4.1 Generation of Markov chains based on pathway constraints

In this section, we will discuss two algorithms to generate Markov chains satisfying the PTIM steady state sensitivities while incorporating the directional pathway structures as emphasized in Section 3.

Each target inhibition combination can be considered as multiplying a matrix *T*_*c*_ to the initial Markov chain *P*. Each row of *T*_*c*_ contains only one non-zero element of 1 based on how the inhibition alters the state. If we consider *n* targets, *n* *T*_*c*_’s in combination can produce a total of 2^*n*^ possible transformation matrices $T_{1},T_{2},\cdots \;,T_{2^{n}}$. The PTIM denotes the stationary state probability of the LSB = 0 for the 2^*n*^ Markov chains ${\text{PT}}_{1},{\text{PT}}_{2},\cdots \;,{\text{PT}}_{2^{n}}$ starting from initial state 11⋯1 (i.e., all kinases considered in the PTIM and tumor are activated). The transition probability matrix has 2^*n*+1^×2^*n*+1^ variables to be inferred and the number of equations available is 2^*n*^. To narrow down the constraints, we will consider the possible BNs that can be generated for each set of possible mutations or outside activations of the thresholded PTIM. Each BN corresponding to a different mutation or initial activation pattern can provide information on possible alterations producing the required PTIM.

#### 4.1.1 4.1.1 Algorithm 1

The first algorithm to generate Markov chains satisfying the PTIM sensitivities is presented in Algorithm ??.





A simulation example for the application of Algorithm ?? is shown next based on the PTIM in Table 2. If we consider a threshold of *α*=0.5 and assuming *K*_1_ and *K*_2_ as initial mutations, the inferred deterministic BN is as shown in Figure 6. Note that the threshold *α* is selected based on the minimum sensitivity considered significant from the perspective of intervention. Since a drug is often considered effective if the concentration to reduce the tumor volume by 50% is within approved dosage, we considered a threshold of 0.5 for normalized sensitivity to denote effectiveness. The threshold should be decreased if we want to incorporate low sensitivity inhibitions in our modeling. To achieve the probabilities shown in Table 2, we apply steps 3 to 7 of Algorithm ?? to generate the Markov chain shown in Table 3. Note that the Markov chain shown in Table 3 is not ergodic and thus the stationary distribution may depend on the starting state. To make the Markov chain ergodic, we can add a small perturbation probability to the Markov chain [20]. The corresponding steady state sensitivities generated by the Markov chain for a perturbation probability *p*=0.001 is shown in Table 4 which closely reflects the PTIM steady state sensitivities shown in Table 2.

**Table 2:** **Example PTIM**

	0 0	0 1	1 1	1 0
0	0	0	0.8	0
1	0.55	0.65	0.9	0.7

**Table 3:** **Example of Markov chain transition probability matrix**

	0 0 0 0	0 0 0 1	0 0 1 0	0 0 1 1	0 1 0 0	0 1 0 1	0 1 1 0	0 1 1 1	1 0 0 0	1 0 0 1	1 0 1 0	1 0 1 1	1 1 0 0	1 1 0 1	1 1 1 0	1 1 1 1
0 0 0 0	0	0.1	0.12	0	0	0	0	0	0	0	0	0	0.78	0	0	0
0 0 0 1	0	0.1	0	0	0	0	0	0	0	0	0	0	0.9	0	0	0
0 0 1 0	0	0	0	0.2	0	0	0	0	0	0	0	0	0	0.8	0	0
0 0 1 1	0	0	0	0.2	0	0	0	0	0	0	0	0	0	0.8	0	0
0 1 0 0	0	0	0	0	0	0.3	0	0	0	0	0	0	0	0	0.7	0
0 1 0 1	0	0	0	0	0	0.3	0	0	0	0	0	0	0	0	0.7	0
0 1 1 0	0	0	0	0	0	0	0	0	0	0	0	0	0	0	0	1
0 1 1 1	0	0	0	0	0	0	0	0	0	0	0	0	0	0	0	1
1 0 0 0	0	0	0	0	0	0	0	0	0	0.35	0	0	0	0	0.65	0
1 0 0 1	0	0	0	0	0	0	0	0	0	0.35	0	0	0	0	0.65	0
1 0 1 0	0	0	0	0	0	0	0	0	0	0	0	0	0	0	0	1
1 0 1 1	0	0	0	0	0	0	0	0	0	0	0	0	0	0	0	1
1 1 0 0	0	0	0	0	0	0	0	0	0	0	0	0	0	0.45	0.55	0
1 1 0 1	0	0	0	0	0	0	0	0	0	0	0	0	0	0.45	0.55	0
1 1 1 0	0	0	0	0	0	0	0	0	0	0	0	0	0	0	0	1
1 1 1 1	0	0	0	0	0	0	0	0	0	0	0	0	0	0	0	1

**Table 4:** **Simulated PTIM from Markov chain**

	0 0	0 1	1 1	1 0
0	0.002003	0.002994	0.800463	0.002995
1	0.549716	0.649251	0.89785	0.698992

#### 4.1.2 4.1.2 Algorithm 2

Another perspective on this issue is based on considering that the tumor is heterogeneous and the observed PTIM response is the aggregate effect of inhibition on multiple clones. The dynamics of each clone can be represented by a BN and there is a small probability *q* of one clone converting to another clone. Thus, the overall system can be represented by a context-sensitive probabilistic Boolean network with perturbation probability *p* and network transition probability *q*[21]. The algorithm to generate a context-sensitive PBN satisfying the observed PTIM behavior is presented as Algorithm ??. Note that based on collapsed steady state probabilities of context-sensitive PBNs [21], Algorithm ?? will always achieve the desired PTIM response within an error of *ε* when *p* and *q* are selected to be small.





**Figure 10:**
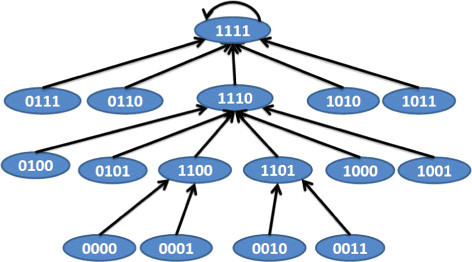
**BN 1 for example 2.**

**Figure 11:**
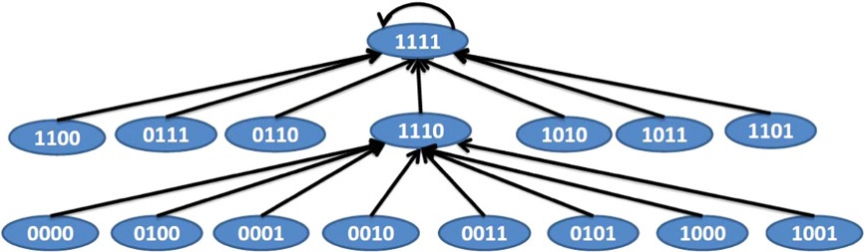
**BN 2 for example 2.**

**Figure 12:**
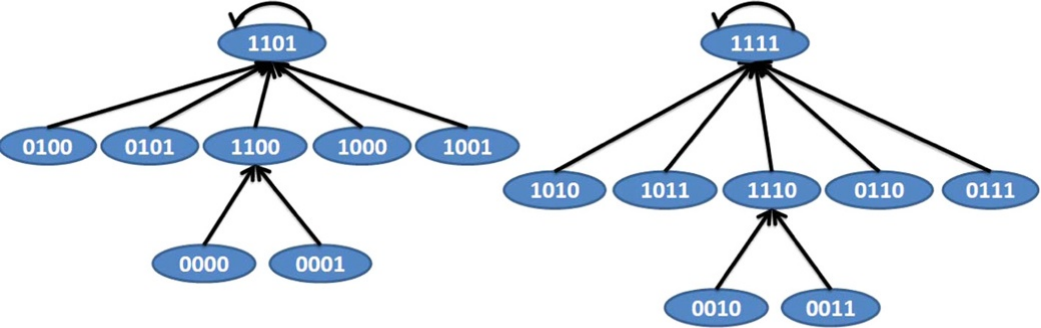
**BN 3 for example 2.**

As an example of application of Algorithm ??, let us consider the PTIM shown in Table 5. Based on Algorithm ?? with *ε*=0.05, we will have three individual BNs BN_1_,*B* *N*_2_,*B* *N*_3_ with selection probabilities of 0.65, 0.25, and 0.1 respectively. The TIMs corresponding to the BNs are shown in Table 6. The BNs satisfying the TIMs in Table 6 are shown in Figures 10, 11 and 12. Using a *p*=0.001 and *q*=0.001, we arrive at the simulated PTIM shown in Table 7 which closely reflects the starting PTIM shown in Table 5.

**Table 5:** **Example PTIM 2**

	0 0	0 1	1 1	1 0
0	0.02	0.01	0.98	0.03
1	0.65	0.89	1	0.9

**Table 6:** **TIMs for the** ***BN***_**1**_ **,** ***BN***_**2**_ **, and** ***BN***_***3***_ **, respectively**

	0 0	0 1	1 1	1 0
BN_1_	0	0	1	0
	1	1	1	1
BN_2_	0	0	1	0
	0	1	1	1
BN_3_	0	0	1	0
	0	0	1	0

**Table 7:** **Simulated PTIM based on Algorithm ?? with** ***p=0.001*** **and** ***q=0.001***

	0 0	0 1	1 1	1 0
0	0.0017	0.0029	0.9964	0.0029
1	0.6495	0.8982	0.9981	0.8982

Note that the dynamical models allow us to generate further insights on possible outcomes with sequential application of drugs. For instance, if we consider the previous example with the inferred context-sensitive PBN generating the PTIM shown in Table 5 and continuously apply a drug *D*_1_ that inhibits *K*_2_ and *K*_3_, we achieve a sensitivity of 0.9. Similarly, continuous application of a drug *D*_2_ that inhibits *K*_1_ and *K*_3_ will generate a sensitivity of 0.9. However, if we alternate the application of *D*_1_ and *D*_2_, we achieve a sensitivity 0f 0.94. It shows that alternate inhibition of these pathways allows us to lower the steady state mass of tumorous states. On the other hand, different sequence of inhibitions can negatively affect the final sensitivity. For instance, if a drug *D*_3_ that inhibits *K*_1_ and *K*_2_ and another drug *D*_4_ that inhibits *K*_3_ is applied alternatively, we achieve a sensitivity of 0.50. Note that *D*_3_ alone produces a sensitivity of 0.99 and *D*_4_ produces a sensitivity of 0.65. This shows that stopping the inhibition of *D*_3_ or *D*_4_ at every alternate step causes the tumor to grow back again. For instance, if no inhibition is applied at every alternate time step, we achieve a sensitivity of 0.49 for *D*_3_ and 0.01 for *D*_4_.

In this section, we presented two algorithms for generation of Markovian models that have inhibition profiles (termed model generated PTIM) similar to our starting PTIM. The motivation behind the two algorithms is based on two widely accepted evolution models of cancer (cancer stem cell model and clonal evolution model [22]) since the primary application of this study is in the context of modeling tumor proliferation pathways. A cancer stem cell model assumes that observed heterogeneity in cancer is due to tumorigenic cancer cells that can differentiate into diverse progeny of cells forming the bulk of tumor [22]. Thus, Algorithm ?? tries to capture this idea of starting with a single network model and altering parts of the model to generate the observed inhibition response. The clonal evolution of cancer model assumes that tumor can consist of multiple clones without hierarchical organization [22]. Thus, Algorithm ?? considers the inhibition response to be based on diverse multiple clones (modeled as separate Boolean networks) with different responses to target inhibitions. The PTIM sensitivity values are used to estimate the network selection probabilities that are similar to proportions of each clone in the heterogeneous tumor. Similar to clonal evolution of cancer model, no single starting network model and its alterations is assumed in Algorithm ?? to generate the stochastic model.

### 4.2 4.2 Biological example

In this example, we consider a PTIM generated from actual biological data and infer a stochastic dynamic network model that produces inhibition responses similar to the experimental PTIM. We consider a canine osteosarcoma tumor sample perturbed with 60 targeted drugs with unique target inhibition profiles to generate steady state cell viability values [14]. Note that available time series data for perturbation studies are mostly for single gene knockouts/knockdowns [23] which are unable to provide the sufficient information to estimate the cell viability response for all possible target inhibition combinations. Thus, due to the absence of time series data and ground truth dynamic networks for drug inhibition studies, our model design criteria is to generate dynamic models that can create the experimentally inferred PTIM while satisfying structural constraints of cancer pathways.

The PTIM generated from experimental 60 drug screen data and satisfying biological constraints [14] for canine tumor sample Sy is shown in Table 8. There are 6 target kinases (IGF1R, PSMB5, TGFBR2, AKT2, EGFR, HDAC1) in this model and the 64 entries in Table 8 refers to the 2^6^=64 possible target inhibitions of the kinases. For instance, second row and seventh column entry of 0.76 refers to sensitivity of 0.76 when the tumor culture is inhibited by IGFR1, TGFBR2, and HDAC1.

**Table 8:** **PTIM generated from a 60 drug screen data for canine tumor sample Sy [**[14]**]**

							IGF1R	IGF1R	IGF1R	IGF1R
					PSMB5	PSMB5	PSMB5	PSMB5		
				TGFBR2	TGFBR2			TGFBR2	TGFBR2	
			0.11	0.38	1	1	1	1	0.68	0.60
		HDAC1	0.55	0.64	1	1	1	1	0.76	0.68
	EGFR	HDAC1	0.64	0.64	1	1	1	1	0.76	0.76
	EGFR		0.17	0.52	1	1	1	1	0.68	0.68
AKT2	EGFR		0.58	0.64	1	1	1	1	0.76	0.76
AKT2	EGFR	HDAC1	0.73	0.76	1	1	1	1	0.88	0.84
AKT2		HDAC1	0.64	0.73	1	1	1	1	0.84	0.76
AKT2			0.47	0.57	1	1	1	1	0.76	0.68

**Figure 13:**
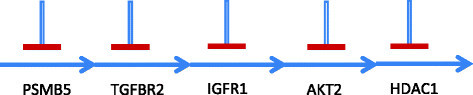
**Directional pathway satisfying TIM of Table** 9 **.**

Considering the overall idea of generation of context-sensitive PBNs, we arrive at the TIM shown in Table 9 using a threshold of 0.3. One of the possible directional pathways that will produce the TIM of Table 9 is shown in Figure 13. Note that there can be multiple other possible directional pathway combinations that can produce the above TIM and we are selecting only one of them with assumed mutation in PSMB5. Further biological data such as gene mutation and expression data and analysis presented in Section 3.1 can be used to narrow down the possible combinations.

**Table 9:** **TIM generated from the PTIM in Table** 8 **using a threshold of 0.3**

							IGF1R	IGF1R	IGF1R	IGF1R
					PSMB5	PSMB5	PSMB5	PSMB5		
				TGFBR2	TGFBR2			TGFBR2	TGFBR2	
			0	1	1	1	1	1	1	1
		HDAC1	1	1	1	1	1	1	1	1
	EGFR	HDAC1	1	1	1	1	1	1	1	1
	EGFR		0	1	1	1	1	1	1	1
AKT2	EGFR		1	1	1	1	1	1	1	1
AKT2	EGFR	HDAC1	1	1	1	1	1	1	1	1
AKT2		HDAC1	1	1	1	1	1	1	1	1
AKT2			1	1	1	1	1	1	1	1

**Figure 14:**
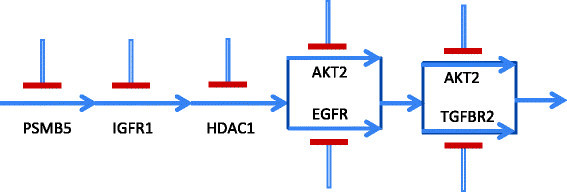
**Directional pathway satisfying TIM of Table** 10 **.**

Subsequently, to select the next level of differences in sensitivities, we considered a threshold of 0.55 which introduces three more possible combinations that fail to stop proliferation (i.e., binarized sensitivity of 0). The TIM is shown in Table 10 and a corresponding directional pathway that produces the TIM is shown in Figure 14. Note that the pathway in Figure 14 requires inhibition of multiple targets as compared to the previous pathway in Figure 13 for stopping tumor proliferation. The first three kinases are the same for the two pathways but the next possibilities are combinations of two kinases rather than single kinase inhibitions.

**Table 10:** **TIM generated from the PTIM in Table** 8 **using a threshold of 0.55**

							IGF1R	IGF1R	IGF1R	IGF1R
					PSMB5	PSMB5	PSMB5	PSMB5		
				TGFBR2	TGFBR2			TGFBR2	TGFBR2	
			0	0	1	1	1	1	1	1
		HDAC1	1	1	1	1	1	1	1	1
	EGFR	HDAC1	1	1	1	1	1	1	1	1
	EGFR		0	0	1	1	1	1	1	1
AKT2	EGFR		1	1	1	1	1	1	1	1
AKT2	EGFR	HDAC1	1	1	1	1	1	1	1	1
AKT2		HDAC1	1	1	1	1	1	1	1	1
AKT2			0	1	1	1	1	1	1	1

**Figure 15:**
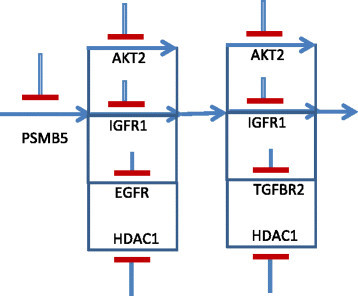
**Directional pathway satisfying TIM of Table** 11 **.**

We next consider a threshold of 0.8 that differentiates the cluster of sensitivity values {0.84,0.84,0.88} from the remaining values. The TIM for this threshold is shown in Table 11, and a corresponding directional pathway that produces the TIM is shown in Figure 15. The directional pathway is more constrained than the previous pathways in having blocks of targets that require more number of inhibitions to stop tumor proliferation.

**Table 11:** **TIM generated from the PTIM in Table** 8 **using a threshold of 0.8**

							IGF1R	IGF1R	IGF1R	IGF1R
					PSMB5	PSMB5	PSMB5	PSMB5		
				TGFBR2	TGFBR2			TGFBR2	TGFBR2	
			0	0	1	1	1	1	0	0
		HDAC1	0	0	1	1	1	1	0	0
	EGFR	HDAC1	0	0	1	1	1	1	0	0
	EGFR		0	0	1	1	1	1	0	0
AKT2	EGFR		0	0	1	1	1	1	0	0
AKT2	EGFR	HDAC1	0	0	1	1	1	1	1	1
AKT2		HDAC1	0	0	1	1	1	1	1	0
AKT2			0	0	1	1	1	1	0	0

Note that the thresholds can be selected in various ways. For instance, we considered equal intervals of 0.25 following the starting threshold of 0.3 resulting in thresholds of 0.3, 0.55, and 0.8. Another approach can be using unequal increment thresholds to maintain sensitivity clusters. Since the experiments conducted to generate the sensitivity information can contain noise, it is preferable to ignore small sensitivity differences.

Once we have the three directional pathways, we used the directional pathway to BN approach of Section 3.2 to generate the Boolean networks BN_1_,*B* *N*_2_, and BN_3_ corresponding to the directional pathways of Figures 13, 14, 15, respectively. Based on the limits of the thresholds, we assigned a selection probability of 0.5 for BN_1_ (0.25<0.5<0.55), 0.25 for BN_2_ (0.55<0.5+0.25<0.8), and remaining 0.25 for BN_3_. Using a value of *p*=0.001 and *q*=0.001, we generated a context-sensitive PBN and calculated the PTIM for the model by generating the steady state probabilities of tumor state = 0 for each target inhibition combination. The generated PTIM for the designed model is shown in Table 12 (up to two decimal digits). The model generated PTIM is similar to our initial experimental PTIM shown in Table 8. The mean and maximum absolute errors of the entries between the experimental and model generated PTIM are 0.043 and 0.2, respectively, which is low considering that only three BNs were used to generate the context-sensitive PBN. Further reduction in the differences between the experimental and model-generated PTIM can possibly be achieved by increasing the number of BNs and optimizing the thresholds and network selection probabilities to reduce the mean error.

**Table 12:** **PTIM generated from context-senstive probabilistic Boolean network model based on Algorithm ??**

							IGF1R	IGF1R	IGF1R	IGF1R
					PSMB5	PSMB5	PSMB5	PSMB5		
				TGFBR2	TGFBR2			TGFBR2	TGFBR2	
			0.00	0.50	0.99	0.99	0.99	1.00	0.75	0.75
		HDAC1	0.75	0.75	1.00	0.99	0.99	1.00	0.75	0.75
	EGFR	HDAC1	0.75	0.75	1.00	0.99	0.99	1.00	0.75	0.75
	EGFR		0.00	0.50	0.99	0.99	0.99	1.00	0.75	0.75
AKT2	EGFR		0.75	0.75	1.00	0.99	0.99	1.00	0.75	0.75
AKT2	EGFR	HDAC1	0.75	0.75	1.00	1.00	1.00	1.00	1.00	1.00
AKT2		HDAC1	0.75	0.75	1.00	0.99	0.99	1.00	1.00	0.75
AKT2			0.50	0.75	1.00	0.99	0.99	1.00	0.75	0.75

## 5Conclusions

In this article, we analyzed the inference of dynamical models from static target inhibition map models. We showed that the inferred blocks from the TIM approach could be converted to directional pathways based on different mutation scenarios and subsequently converted to dynamic BN models. In terms of stochastic model inference, we presented two algorithms where (i) the first technique was based on altering the BN generated from binarizing the PTIM based on a single threshold and (ii) the second approach considered as generation of multiple BNs based on different thresholds and integrating them in the form of a context-sensitive PBN. We provided examples to show the application of the algorithms to generate Markovian models whose steady state inhibition profiles are close to the experimental PTIMs.

Note that the inference algorithms designed in this article are primarily focused on dynamic models of tumor proliferation. The number of targets considered is small as they are a subset of the targets of targeted drugs (usually tyrosine kinase inhibitors) that are required to faithfully capture the tumor proliferation of a particular system without overfitting. Consequently, any properties of large-scale genetic regulatory networks [23],[24] such as adherence to power law [25] were not incorporated in these studies. Future studies will try to explore the incorporation of characteristics of large-scale networks in inference of dynamic models from PTIMs. The PTIM can be considered as a model expressing the relative sensitivity of the tumor proliferation following inhibition. If we consider the definition of relative expression level variation (RELV) [26] as $\frac{\eta_{\text{ij}}}{x_{i}^{\text{wt}}}$ where *η*_*ij*_ is the steady state expression level variation of gene *i* after the knockout/knockdown of gene *j* and $x_{i}^{\text{wt}}$ is the expression level of gene *i* in wild type, a corresponding analogous sensitivity mapping can be derived by replacing $x_{i}^{\text{wt}}$ by cell viability without any inhibition and *η*_*ij*_ being replaced by change in cell viability following inhibition *j*. Here, *j* consists of 2^*T*^ combinations for *T* targets as compared to *T*+1 knockouts usually considered in RELV analysis. For individual protein targets in the binary deterministic BN models, the RELVs can be mapped to the relative change in the attractor states of *P* and *P* *T*_*j*_ where *P* denotes the transition matrix for the BN without inhibition and *P* *T*_*j*_ denotes the transition matrix following inhibition *j*. The binarization of the different proteins will be based on different thresholds based on their relative behavior. Similarly, for the Markov chain model, the relative change in the steady state probabilities of expressed protein *i* in *P* and *P* *T*_*j*_ will be analogous to RELV. Note that the binary deterministic and stochastic formulation employed in our analysis incorporates the relative sensitivity behavior that has been earlier observed to be more appropriate for regulatory network inference [26].

Future research will involve analyzing mutation data to restrict the possible directional pathways along with time series experimentation for inference of the unique dynamic model.

## Authors’ original submitted files for images

Below are the links to the authors’ original submitted files for images. Authors’ original file for figure 1 Authors’ original file for figure 2 Authors’ original file for figure 3 Authors’ original file for figure 4 Authors’ original file for figure 5 Authors’ original file for figure 6 Authors’ original file for figure 7 Authors’ original file for figure 8 Authors’ original file for figure 9 Authors’ original file for figure 10 Authors’ original file for figure 11 Authors’ original file for figure 12 Authors’ original file for figure 13 Authors’ original file for figure 14 Authors’ original file for figure 15 Authors’ original file for figure 16 Authors’ original file for figure 17
